# Healthcare outcomes in undocumented immigrants undergoing two emergency dialysis approaches 

**DOI:** 10.5414/CN109137

**Published:** 2017-08-17

**Authors:** S. Jawad Sher, Waqas Aftab, Ranjani N. Moorthi, Sharon M. Moe, Christopher S. Weaver, Frank C. Messina, Nancy M. Martinez-Hoover, Melissa D. Anderson, Michael T. Eadon

**Affiliations:** 1 Division of Nephrology and; 2 Department of Emergency Medicine, Indiana University School of Medicine, Indianapolis, IN, USA

**Keywords:** undocumented immigrant, end-stage renal disease, dialysis, InterQual, safety-net hospital

## Abstract

Background: Current estimates suggest 6,500 undocumented end-stage renal disease (ESRD) patients in the United States are ineligible for scheduled hemodialysis and require emergent dialysis. In order to remain in compliance with Emergency Medicaid, an academic health center altered its emergency dialysis criteria from those emphasizing interdialytic interval to a set emphasizing numerical thresholds. We report the impact of this administrative change on the biochemical parameters, utilization, and adverse outcomes in an undocumented patient cohort. Methods: This retrospective case series examines 19 undocumented ESRD patients during a 6-month transition divided into three 2-month periods (P1, P2, P3). In P1, patients received emergent dialysis based on interdialytic interval and clinical judgment. In P2 (early transition) and P3 (equilibrium), patients were dialyzed according to strict numerical criteria coupled with clinical judgment. Results: Emergent criteria-based dialysis (P2 and P3) was associated with increased potassium, blood urea nitrogen (BUN), and acidosis as compared to P1 (p < 0.05). Overnight hospitalizations were more common in P2 and P3 (p < 0.05). More frequent adverse events were noted in P2 as compared to P1 and P3, with an odds ratio (OR) for the composite endpoint (intubation, bacteremia, myocardial infarction, intensive care unit admission) of 48 (5.9 – 391.2) and 16.5 (2.5 – 108.6), respectively. Per-patient reimbursement-to-cost ratios increased during criteria-based dialysis periods (P1: 1.49, P2: 2.3, P3: 2.49). Discussion: Strict adherence to criteria-based dialysis models increases biochemical abnormalities while improving Medicaid reimbursement for undocumented immigrants. Alternatives to emergent dialysis are required which minimize cost, while maintaining dignity, safety, and quality of life.

Supplemental material is available for free at: http://www.dustri.com Clinical Nephrology, Vol. 88; October, 2017

## Introduction 

The current number of undocumented immigrants residing in the United States of America is estimated at 11 – 12 million [1]. Provision of healthcare for these individuals is an ongoing challenge. Healthcare costs for this population result in an annual burden of 4.3 billion dollars in additional spending by city, state, and federal governments [2]. Undocumented end-stage renal disease (ESRD) patients require indefinite hemodialysis (HD), posing a longitudinal financial burden to health systems nationwide [3]. 

Federal law explicitly denies healthcare-related benefits to immigrants who are undocumented or who have not met residency requirements, leaving such decisions to states that rely largely on emergency Medicaid (E-Medicaid) funds to cover these costs. Additionally, the Affordable Health Care Act of 2010 (ACA) does not support regular outpatient dialysis care for undocumented immigrants [4, 5]. Further, planned reductions in E-Medicaid funding are predicted [6, 7], potentially forcing individual hospital systems to absorb the heavy financial burden of treating undocumented ESRD patients [8]. This practice risks the solvency of the safety-net hospitals these patients often depend upon and may increase costs for the general population. In Indiana, E-Medicaid reimbursement from the Centers of Medicare and Medicaid Services (CMS) is impacted by corporate emergency treatment criteria [9], such as the McKesson InterQual^®^ criteria [10, 11]. These corporate criteria do not delineate explicit guidelines to provide emergent dialysis to ESRD patients. Faced with this dilemma, some safety-net health centers have developed internal criteria to help inform physicians whether undocumented ESRD patients meet a threshold to receive emergency dialytic care [12, 13]. In these models, patients receive dialysis only after meeting the CMS definition of an emergency medical condition [14], frequently demonstrating life-threatening sequelae of their ESRD. 

A well-organized, clearly-demarcated transition to a model based upon strict emergent dialysis criteria occurred in an academic center’s safety-net hospital. This transition was not a clinical study; instead, the transition was administratively implemented by the health system in order to remain in regulatory compliance with Indiana Emergency Medicaid Services. Before and after the transition, up to 19 patients presented as needed to the emergency department (ED) for emergency dialysis admissions because outpatient dialysis was unavailable for these patients in the health system. While all admissions met inpatient InterQual^®^ criteria, the health system emphasized different criteria to guide clinicians before and after the transition. Prior to the transition, patients were provided emergent dialysis based on clinical judgment and an interdialytic interval equal to or exceeding 3 days. Most patients received intermittent dialysis twice weekly at consistently spaced intervals, and were discharged following dialysis. On a predetermined date, patients were sharply transitioned to a care model in which clinicians were guided by numerical criteria developed by the health system. Criteria such as laboratory data and oxygen saturation levels were emphasized. Patients meeting criteria were admitted for dialysis. Patients were discharged without dialysis if they failed to meet criteria and clinicians agreed that an immediate threat to life was not present. 

For 19 patients, we report the short-term medical and financial implications of this transition from criteria based on interdialytic interval (essentially twice weekly dialysis) to numerical thresholds. 

## Materials and methods 

### Study design 

This is a retrospective case series of 19 adult undocumented ESRD patients who received emergent dialysis from an academic health system during a 6-month transition. The study was approved by the Institutional Review Board (IRB) of the university and safety-net hospital system. 

### Periods 

The 6-month transition was divided into three distinct 2-month periods (P1, P2, and P3) listed in Table 1. Period 1 (P1) corresponds to the 2 months immediately preceding the transition wherein patients received intermittent dialysis twice weekly and were discharged following dialysis. On a predetermined date, all patients were sharply transitioned from P1 to a care model based on emergency dialysis criteria developed by the health system (Supplemental Document 1). Period 2 (P2) and period 3 (P3) represent the 4 consecutive months following the transition. P2 corresponds to the “early transition,” immediately following the transition. P3 followed P2 and represents an “equilibrium phase”. In clinical practice, no sharp demarcation existed between P2 and P3; however, the authors divided these periods because considerable changes in the behavior of patients and practitioners were noted as they adjusted to the new criteria. For example, far fewer patients were discharged without dialysis in P3, perhaps related to patients’ better understanding of admission requirements and providers’ increased comfort in exercising clinical judgment to supersede the numerical criteria. At the rounding nephrologist’s discretion, patients in P2 and P3 often required serial dialysis with multiple-day admissions to improve clinical status, while reducing the dialysis disequilibrium risk. 

Before the transition, protocols were established for patient triage, admission, and discharge based on the new emergency dialysis criteria. Patients were given written information about the forthcoming changes, provided legal counsel to aid in changing immigration status, and offered travel assistance to transition to a state or country with scheduled dialysis options. Counselors educated patients on uremic symptoms and nutrition. However, the transition was not a prospective study and this retrospective investigation was not a pre-specified component of the transition. This study began as a fellow’s continuous quality improvement (CQI) project in response to the transition. 

### Patient selection 

Patients were included in the study analysis if all dialysis sessions in at least one period (P1, P2, P3) were performed exclusively at the safety-net hospital (N = 19). Patients’ dialysis location for every treatment was verified using the Indiana Health Information Exchange, an internet system linking medical records for most health systems across the state of Indiana. Patients were excluded from statistical analyses for a given period if they did not receive dialysis in our health system or received dialysis in another health system during that period. 

### Data collection 

Demographic, laboratory, resource utilization, and outcome data were collected by chart review. Factors collected include: immigration status, dialysis vintage, dialysis access, dialysis session quantity, hours of dialysis received, interdialytic interval, number of ED visits, electrocardiograms (ECGs), chest radiographs, hospital nights crossing midnight, intensive care unit (ICU) days, average presenting blood pressure (BP), peak BP, average blood urea nitrogen (BUN), peak BUN, average potassium, peak potassium, average calcium, phosphorus, pre-dialysis sodium, weight, albumin, parathyroid hormone (PTH), and hemoglobin. Peak refers to the highest recorded value in a period, average is the mean of all values in a period. Adverse events from dialysis or lack of dialysis were recorded. Major adverse events included respiratory failure, bacteremia, ICU admission, and myocardial infarction (MI). Minor adverse events included blood transfusions, dialysis disequilibrium, and interventional radiology (IR) procedures. No patients died during the study periods. 

### Charge, cost, and reimbursement data 

Charge data were estimated according to both a basic and complex model. The models included hospital facility fees and location-specific charge data from the American Medical Association’s (AMA) Fair Health Consumer cost lookup (Supplemental Table 1) (http://fairhealthconsumer.org/medicalcostlookup.php). The basic model included facility fees for ICU stays, nights hospitalized, ED visits, and dialysis sessions. The complex model included the basic model components as well as fees for chest radiographs, ECGs, laboratory testing, angioplasties, catheter exchanges, transfusions, and physician charges of the ED physicians, intensivists, hospitalists, and nephrologists. Costs and reimbursements to the hospital system were obtained. Charges, costs, and reimbursements from P1, P2, and P3 were compared. 

### Statistical analysis 

Statistical analysis was performed using SigmaStat 3.5 and GraphPad Prism 4.0. ANOVA and Kruskal-Wallis tests were used to compare normally and non-normally distributed data, respectively. Data is presented as mean ± standard deviation or median (interquartile range). A multiple corrections testing penalty was applied with Holm-Sidak methodology. Thresholds of significance are p < 0.017 (first comparison), p < 0.025 (second comparison), and p < 0.05 (third comparison). Categorical variables were compared with an odds ratio (OR) and 95% confidence interval (CI). p < 0.05 was considered significant. 

## Results 

### Baseline characteristics 

Of the 19 undocumented ESRD patients, the mean age was 36.6 years, the mean dialysis vintage was 20.2 months, and 74% had a functional arteriovenous fistula (Table 2, Supplemental Table 2). 18 patients received all of their dialysis sessions at the safety-net hospital during P1. Five of these left the cohort in early P2 and 1 returned in P3. The 19^th^ patient was not present all of P1, but was present during P2 and P3. Thus, there were 18 patients in P1, 14 in P2, and 15 in P3. The main factors in attrition were immigration status change and transfer to another hospital system. The total number of ED encounters resulting in discharge without dialysis was assessed for the cohort. In P1, 1 encounter resulted in discharge from the ED without dialysis. In P2, 17 encounters resulted in discharge without dialysis as compared to P3, when 3 visits failed to result in dialysis. ED presentations unrelated to ESRD symptoms were excluded. 

### Biochemical abnormalities and dialysis adequacy 

Table 3 summarizes the significant biochemical abnormalities across periods. The average of each patient’s peak serum potassium level and mean potassium level varied significantly between periods (p = 0.003 for peak and p = 0.033 for mean). Patients receiving dialysis based on numerical criteria (P2 and P3) had higher serum potassium levels than P1. Potassium values for hemolyzed blood samples were excluded. Mean serum bicarbonate levels varied across periods (p < 0.001), with lower levels during P2 and P3 as compared to P1 (p < 0.001 for both comparisons). Both the average of each patient’s highest BUN level and the average of each patient’s mean BUN level varied across periods (p = 0.015 and 0.007, respectively). BUN levels were greater in P2 and P3 compared to P1. No differences in potassium, bicarbonate, or BUN were noted between P2 and P3. Blood pressure, weight, sodium, hemoglobin, calcium, phosphorus, and PTH levels remained unchanged across periods. 

The maximum interdialytic interval, quantity of dialysis sessions, and total hours of dialysis were determined. The median maximum interdialytic interval differed between periods (p < 0.001). The interval was shortest in P1 (4 days) and longer during P2 (8 days) and P3 (9 days), with p < 0.001 for both comparisons. In contrast, the dialysis session quantity did not change across periods (P1: 17.0 ± 4.1, P2: 16.9 ± 4.4, P3: 15.9 ± 4.7), as nephrologists often provided serial dialysis treatments during P2 and P3. To prevent disequilibrium, some patients in P2 and P3 received abbreviated initial treatments upon admission. The median hours of dialysis varied between periods (p = 0.037). Total dialysis time was lower in P2 (58, 44 – 68 hours) compared to P1 (72, 68 – 72 hours), p = 0.006. Although the median of dialysis hours during P3 (57, 39 – 76 hours) was similar to P2, the range was wider and not significantly different than either P1 or P2. 

### Adverse events 

Major adverse events included respiratory failure, bacteremia, MI, and ICU admission. No patients died during the study. 26 major events were observed (Table 4). The majority occurred in P2 (65%), followed by P3 (27%) and P1 (8%). A composite endpoint including respiratory failure, bacteremia, non-ST-elevation myocardial infarction (NSTEMI) and ICU admission occurred most frequently during P2 (85.7%), followed by P3 (26.7%) and P1 (11%). The odds of meeting this composite endpoint in P2 as compared to P1 was 48.0 (95% CI, 5.9 – 391.2). While the biochemical abnormalities of P2 and P3 closely mirrored each other, fewer adverse events were observed in P3. The OR of meeting the composite endpoint in P2 compared to P3 was 16.5 (2.5 – 108.6). No difference was observed between P1 and P3. A composite endpoint of respiratory failure, bacteremia, and NSTEMI (excluding ICU admission) was also examined. The OR for P2 compared to P1 was 12.8 (95% CI, 1.3 – 124.4). No significant difference was noted for other comparisons. 

21 minor adverse events occurred, including blood transfusions, disequilibrium syndrome, and access related complications requiring catheter exchange, fistulogram, or angioplasty. Minor adverse events occurred most frequently in P2 (38%), followed by P1 (30%) and P3 (28.57%). A composite OR of minor event occurrence between P1 and P2 was marginally significant at 4.7 (95% CI, 1.01 – 21.7; p = 0.05). Other OR comparisons were not significant. 

### Utilization 

Healthcare utilization markers are summarized in Table 3. The median number of nights hospitalized was different between periods (p < 0.001), as it increased from 0 (0 – 0) in P1 to 13 (11 – 15) in P2 and 11 (9 – 13) in P3 (p < 0.001 for P2 and P3 vs. P1). In P1, only 3 patients were hospitalized overnight. However, 1 was hospitalized for severe *Clostridium difficile* colitis between day 1 and day 60 of P1, because he was ineligible for nursing facility placement. 

The number of days spent in the ICU varied across periods (p = 0.001). Median days of ICU status increased in P2 (3 days) compared to P1 (0 days) and P3 (0 days), p = 0.001 and 0.009, respectively. P3 did not differ significantly from P1. The number of ED visits and admissions both varied across periods (p < 0.001 for both), decreasing significantly during P2 and P3 as compared to P1 (p < 0.001 for all comparisons to P1), as patients no longer presented to the ED twice weekly for intermittent dialysis. To measure radiology and ancillary service engagement, the number of chest radiographs and ECGs obtained per period were assessed and found to be different (p < 0.001 for both ANOVA tests). Chest radiograph and ECG acquisition increased in P2 and P3 compared to P1 (p < 0.001 for both comparisons). P2 and P3 did not differ in number of nights hospitalized, ED visits, admissions, chest radiographs, or ECGs. 

**Figure 1.**
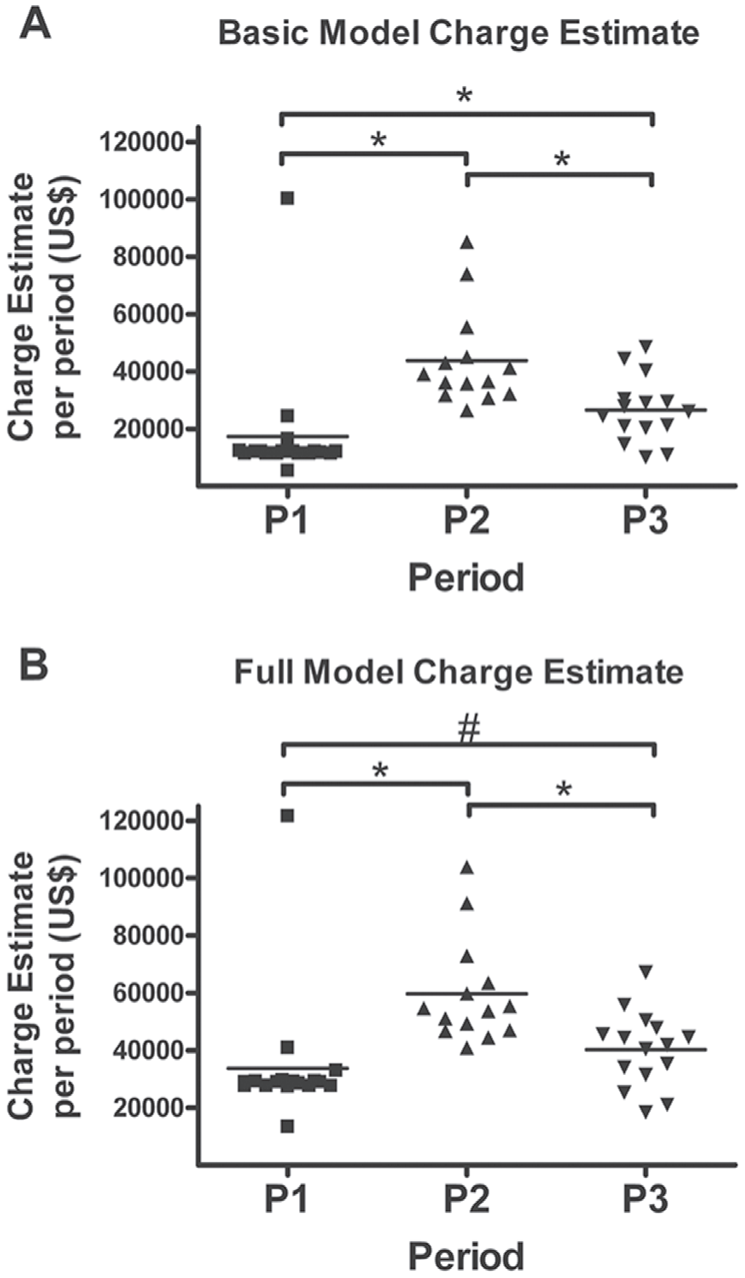
Charge estimates increase during P2. A: Participants receiving dialysis in P2 had significantly greater charge estimates as compared to P1 and P3 according to a basic model estimate of facility fees. Patients also had greater charge estimates during P3 than P1. The median in P1 was $12,024 ($11,356 – 12,182), in P2 was $37,787 ($31,934 – 50,280), and in P3 was $26,516 ($20,255 – 30,267). B: According to the full model estimate including facility and physician fees, patients receiving dialysis during P2 had greater charge estimates than they did during P1 and P3. Changes observed between P1 and P3 were only significant if an outlier in P1 is excluded (#). The median P2 charge estimates were $54,148 ($46,771 – 68,220) as compared to P1 at $28,998 ($27,544 – 29,497). The P3 median charge estimate was $41,840 ($31,387 – 47,742). Column lines represent median values. Estimates are per patient per 2-month period. *Statistically significant by Kruskal-Wallis after multiple testing correction.

To determine the economic impact of these changes, charge estimates were assessed according to two models (Figure 1). In the basic and full models, charge estimates per patient varied between periods (p <  0.001). All basic model individual comparisons were statistically significant, with median P2 charge estimates exceeding those of P1 and P3. P3 estimates also exceeded P1. In the full model which includes physician fees, P2 estimates were $54,148 per patient per period compared to $28,998 in P1 (p = 0.0015). In the full model, P3 did not differ from P1. 

Actual aggregate charge, cost, and reimbursement data for the cohort were also obtained from the health system (Table 5), but do not include physician fees. Total per-patient charges and costs were similar across periods at ~ $75,000 and $14,000, respectively. In contrast, per patient reimbursements increased from $20,309 in P1 to $33,329 in P3. The reimbursement-to-cost ratio favored the emergency criteria systems over the provision of twice weekly dialysis (1.49 in P1, 2.3 in P2, 2.49 in P3). Analogously, the charge-to-reimbursement ratio also favored emergency criteria-based dialysis (3.93 in P1, 2.34 in P2, 2.24 in P3). 

## Discussion 

In some US states, safety-net hospitals bear the burden of dialytic care for undocumented immigrants because federal laws exclude these patients from government healthcare. Despite great interest from the Nephrology community [4, 8, 15], the approach to undocumented immigrant care remains controversial. This study analyzes a case series of undocumented ESRD patients exposed to two different dialytic care models. Neither model is ideal. The significance is in understanding the consequences of transitioning patients from intermittent dialysis to strict criteria-based emergent dialysis. 

An unheralded success of the transition was the health system’s ability to facilitate legal immigration status for several patients prior to the start of P1 through careful case management and legal assistance. To accomplish this, the health system partnered with a community attorney specializing in immigration issues. She assisted 3 patients with securing outpatient dialysis chairs. One patient renewed an expired green card and 2 other patients obtained asylum, allowing all 3 to qualify for public funding. The attorney is currently working with 4 more patients seeking legal citizen status: 1 related to her son being a citizen; 2 through the visa process; and 1 additional patient seeking asylum. 

The most remarkable contrast lies between P1 and P2. In P2, where objective laboratory and oxygen saturation levels were principally considered, the patients’ biochemical abnormalities were of greater severity, adverse events were more frequent, and charge estimates increased. These results parallel those of non-adherent chronic hemodialysis patients [16]. By the “equilibrium phase” of P3, patients and providers had adjusted to the new criteria. Five-fold fewer patients were discharged without dialysis, and fewer adverse events occurred in P3 compared to P2. 

The comparison between P1 and P3 highlights two methodological approaches to undocumented dialytic care. Neither the quantity of dialysis sessions nor the frequency of adverse events differed significantly between periods. The major difference between P1 and P3 was that treatments were intermittent in P1 and grouped in P3. Twice weekly dialysis in P1 was associated with more frequent ED presentations and admissions (by definition). However, dialysis in P3 was associated with longer interdialytic intervals and greater severity of biochemical abnormalities. The more aberrant laboratory values of P3 were required for admission, but resulted in more frequent overnight hospital stays for serial dialysis. Thus, the P1-to-P3 comparison is essentially that of even-spaced intermittent vs. bolus dialysis therapy. Although adverse events did not differ between P1 and P3 in this short study, the long-term implications of hyperkalemia, uremia, and acidosis seen during P3 are associated with poorer outcomes in ESRD patients [17, 18, 19, 20, 21, 22]. Furthermore, undocumented patients excluded from scheduled dialysis repeatedly experience debilitating symptoms and psychosocial distress [23]. 

The findings of our investigation mirror those of other centers. As in New York, our cohort was comprised of young immigrants of Hispanic ethnicity [24]. An academic center in Texas reported similar increases in utilization and cost, with lower Kt/V in immigrants provided hemodialysis emergently as opposed to immigrants receiving thrice weekly dialysis [25]. A report from Israel revealed similar biochemical abnormalities in 15 undocumented immigrants [26]. Our study is unique because it follows the same cohort of patients as it transitioned from consistently spaced dialysis to emergent. It also updates previously reported financial implications [25] for the post-ACA era [27]. 

A prior investigation documented cost savings in transitioning to case manager-based care models [12]. In the present investigation, the charge-to-reimbursement and reimbursement-to-cost ratios favored the P3 care model despite increased hospitalization and utilization. One explanation is patients receiving intermittent twice weekly dialysis often do not meet thresholds for E-Medicaid reimbursement, even though a physician determined the need for inpatient admission. On a per-patient basis, there were no appreciable cost savings or losses during P3 compared to P1. Our median charge estimate data revealed differences between periods in contrast to the aggregate mean charge data of the hospital system. Reasons for this discrepancy may include outliers, small sample size, the inclusion of physician fees, and the fact that our charge estimates utilized usual and customary rates from the AMA (which may not reflect actual hospital system charges). 

The main limitations of this study are its retrospective nature, small sample size, and high attrition rate. The sample size and high attrition rate limit conclusions, but the sample size was adequate to appreciate changes in factors with large effect sizes like adverse outcomes and charge estimates. The 2-month time periods were short, but selected to evenly match the length of the early transition phase of P2. Additional limitations include the use of surrogate dialysis adequacy markers since we could not calculate Kt/V and the high fistula rate which limits generalizability to other centers with higher catheter rates. 

In summary, even carefully planned transitions to criteria-based emergency dialysis care models will require refinement. If health systems are required to change their undocumented dialysis care model, they should avoid a P2 phase. Current CMS reimbursement patterns are linked to InterQual^®^ guidelines [9]. Subtle signs of volume overload and uremia are inadequately conveyed by guidelines. These guidelines, or any others devised to meet the CMS definition of an “emergency medical condition,” cannot substitute for clinical judgment to predict adverse events. In the short term, hospitals may expect improved reimbursement with strict adherence to emergency criteria. However, consequences of under-dialysis preclude extrapolation of these financial data in the long term. Better alternatives to emergent dialysis are required to minimize societal cost, while maintaining dignity, safety, and quality of life in patients. Ultimately, these concerns may only be alleviated by comprehensive immigration reform and cost-effective CMS coverage for all dialysis-dependent patients. A lack of data regarding undocumented ESRD care has been cited as a reason for the slow pace of governmental reforms [15]. We hope the data presented in our study adds additional information to help advance these critical reforms. 

## Funding 

MTE was supported by the PhRMA foundation (Clinical Pharmacology Young Investigator Award), the Norman S. Coplon Satellite Health Extramural Grant Program, and NIH/NIDDK 1K08DK107864-01. 

## Conflict of interest 

The authors have nothing to disclose. 

**Table 1.** Description of periods.

Period	Description of emergency dialysis criteria
P1	Pre-transition: Dialysis based on clinical judgment and an inter-dialytic interval equal to or exceeding 3 days. Admissions were initiated through the Emergency Department with same-day discharge.
P2	Early transition: Dialysis only when meeting numerical criteria (Supplemental Document 1). Patients frequently required serial dialysis treatments after admission to improve electrolyte and volume status.
P3	Equilibrium: Dialysis for the same numerical criteria as P2, but fewer discharges without dialysis occurred in this period. Patients received serial dialysis treatments after admission.

**Table 2.** Key demographics.

Patient characteristic	Value^a^
Age in years on May 1, 2014, mean (range)	36.6 (22 – 50)
Dialysis vintage in months, mean (range)	20.25 (2 – 51.5)
Female gender, N (%)	6 (32)
Employed, N (%)	9 (47)
Married	11 (58)
US citizens	0
Number of children, mean (range)	2.3 (0 – 6)
Mexican, N (%)	14 (74)
Hypertensive, N (%)	17 (89)
Diabetic, N (%)	5 (26)
Arterio-venous fistula as access, N (%)	14 (74)

^a^Mean (range) or number (percent) of total.

**Table 3.** Mean or median statistics by period for all patients.

Clinical factor	P1	P2	P3	p-value^a ^ P1 vs. P2	p-value^a ^ P1 vs. P3	p-value^a ^ P2 vs. P3
Average BP (mmHg)	152.6 ± 20.1	149.8 ± 22.6	156.2 ± 16.3	0.76	0.487	0.33
Peak BP (mmHg)	173.5 ± 25.4	163.7 ± 25.2	178.8 ± 19.3	0.13	0.472	0.025
Average BUN^c^ (mg/dL)	86. 1 ± 1.6	111.6 ± 31.6	113.4 ± 29.8	**0.006**	**0.003**	0.86
Peak BUN^c^ (mg/dL)	113.1 ± 27	137.5 ± 32.6	139.8 ± 27.0	**0.02**	**0.007**	0.58
Average potassium^c^ (mEq/L)	5.1 ± 0.4	5.7 ± 0.9	5.5 ± 0.8	**< 0.001**	**0.002**	0.35
Peak potassium^c^ (mEq/L)	5.9 ± 0.6	6.7 ± 1.2	6.4 ± 1.1	**0.01**	**0.02**	0.25
Average calcium (mg/dL)	8.3 ± 0.9	8.2 ± 0.8	8.7 ± 0.5	0.76	0.10	0.30
Average phosphorus (mg/dL)	6.6 ± 1.7	7.1 ± 1.9	6.7 ± 1.9	0.54	0.93	0.56
Average PTH^b^ (pg/dL)	587 (395 – 760)	479 (362 – 810)	642 (372 – 935)	0.38	0.76	0.32
Average bicarbonate^c^ (mEq/L)	21.4 ± 1.7	16.9 ± 3.7	17.6 ± 4.0	**< 0.001**	**< 0.001**	0.63
Average sodium (mEq/L)	136.3 ± 1.7	135.7 ± 2.1	136.0 ± 1.7	0.42	0.630	0.40
Average hemoglobin (g/dL)	9.8 ± 1.5	9.2 ± 1	9.1 ± 1.1	0.11	0.10	0.81
Peak weight (kg)	68.6 ± 13.9	76.6 ± 20.1	72.5 ± 18.5	0.55	0.59	0.19
Lowest weight (kg)	64.8 ± 15.5	67.5 ± 18.5	65.2 ± 18	0.11	0.065	0.071
Maximum inter dialytic interval^b,c^ (d)	4 (4 – 4)	8 (6 – 9.5)	9 (6 – 10.75)	**< 0.001**	**< 0.001**	0.74
# of HD sessions	17 ± 4.1	16.9 ± 4.4	15.9 ± 4.7	0.93	0.49	0.59
# of hours of dialysis^b,c^	72 (68 – 72)	56 (44 – 65)	56 (45 – 73)	**0.006**	0.13	0.78
# of ED visits^b,c^	17 (17 – 18)	8 (4 – 9)	8 (5 – 9)	**< 0.001**	**< 0.001**	0.97
# of nights in hospital^b,c^	0 (0 – 0)	13 (11.5 – 15)	11 (9 – 13)	**< 0.001**	**< 0.001**	0.057
# of admissions^c^	15.4 ± 5.2	5.9 ± 3.1	7.1 ± 3.0	**< 0.001**	**< 0.001**	0.27
# of days with ICU status^b,c^	0 (0 – 0)	3 (0.5 – 6)	0 (0 – 0)	**0.001**	0.16	**0.009**
# of CXR^c^	0.6 ± 1.1	5.6 ± 2.5	5.3 ± 2.1	**< 0.001**	**< 0.001**	0.86
# of ECG^c^	0.9 ± 1.4	8.4 ± 2.7	8.8 ± 3.1	**< 0.001**	**< 0.001**	0.54

BP = blood pressure; BUN = blood urea nitrogen; PTH = parathyroid hormone; HD = hemodialysis; ICU = intensive care unit; CXR = chest X-ray; ECG = electrocardiogram. Mean ± SD or median (interquartile range) is presented for normally distributed and skewed data, respectively. ^a^Unadjusted p-values between comparisons are provided. When not noted, ANOVA was performed between groups. ^b^Indicates a Kruskal-Wallis test was performed. ^c^Indicates the ANOVA or Kruskal-Wallis study was significant with p < 0.05. Multiple corrections testing is applied with Holm-Sidak methodology. Thresholds of significance are p < 0.017 (first comparison), p < 0.025 (second comparison), and p < 0.05 (third comparison). Significant values are bolded. Conversion factors for urea nitrogen in mg/dL to mmol/L, ×0.357.

**Table 4.** Clinical endpoints.

				P1 vs. P2		P1 vs. P3		P3 vs. P2	
Adverse events	P1, N (%)	P2, N (%)	P3, N (%)	OR (95% CI)	p	OR (95% CI)	p	OR (95% CI)	p
Major AE	N = 18	N = 14	N = 15						
Composite endpoint	2 (11)	12 (86)	4 (27)	**48.0 (5.9 – 391.2)**	**< 0.001**	2.9 (0.5 – 18.7)	0.26	**16.5 (2.5 – 108.6)**	**0.004**
Composite, no ICU	1 (6)	6 (42)	4 (27)	**12.8 (1.3 – 124.1)**	0.029	6.2 (0.6 – 62.8)	0.12	2.06 (0.4 – 9.8)	0.36
Respirary failure	0	2 (14)	1 (7)	7.4 (0.3 – 167.6)	0.21	3.8 (0.1 – 101.1)	0.42	2.3 (0.2 – 29.0)	0.51
Bacteremia	1 (6)	3 (21)	1 (7)	4.6 (0.4 – 50.4)	0.21	1.2 (0.1 – 21.2)	0.89	3.8 (0.4 – 42.0)	0.27
NSTEMI	0	1 (7)	2 (13)	4.1 (0.2 – 108.9)	0.40	6.9 (0.3 – 154.6)	0.23	0.5 (0.0 – 6.22)	0.59
ICU admission	1 (6)	11 (79)	3 (20)	**62.3 (5.7 – 678.2)**	**< 0.001**	4.3 (0.4 – 46.0)	0.23	**14.7 (2.4 – 88.5)**	**0.003**
Minor AE									
Composite endpoint	4 (22)	8 (57)	5 (33)	4.7 (1.01 – 21.7)	0.05	1.8 (0.4 – 8.2)	0.48	0.4 (0.1 – 1.7)	0.20
Blood transfusion	3 (17)	5 (35)	2 (13)	2.8 (0.5 – 14.5)	0.23	0.8 (0.11 – 5.3)	0.79	3.6 (0.6 – 22.9)	0.17
Disequilibrium	1 (6)	1 (7)	1 (7)	1.3 (0.1 – 23.0)	0.85	1.2 (0.1 – 21.2)	0.89	1.1 (0.06 – 19.1)	0.96
IR procedure need	3 (17)	2 (14)	3 (20)	0.8 (0.1 – 5.8)	0.85	1.3 (0.2 – 7.4)	0.81	0.7 (0.1 – 4.7)	0.69

AE = adverse event; ICU = intensive care unit; NSTEMI = non-ST-elevation myocardial infarction; IR = interventional radiology.

**Table 5.** Costs and reimbursements.

Economic factor^a^	P1	P2	P3
Per-patient charges (excludes physician charges)	79,885	77,140	74,687
Per-patient cost (excludes physician fees)	13,652	14,319	13,404
Per-patient reimbursement	20,309	32,959	33,329
Charge-to-reimbursement ratio	3.93	2.34	2.24
Reimbursement-to-cost ratio	1.49	2.3	2.49

^a^These data were provided from the health system as composite data for the entire cohort. Individual patient data is unavailable. Data are presented as means per patient per 2-month period. Formal statistical analysis is precluded.
